# Efficacy and Safety of a Polytetrafluoroethylene Membrane Wrapped a Single Layer of Sirolimus-Eluting Stent in a Porcine Coronary Perforation Model

**DOI:** 10.31083/j.rcm2307233

**Published:** 2022-06-24

**Authors:** Yi Liu, Jingyu Zhou, Xiaoming Wang, Chao Gao, Fangjun Mou, Wangwei Yang, Rutao Wang, Ling Tao

**Affiliations:** ^1^Department of Cardiology, Xijing Hospital, Fourth Military Medical University, 710032 Xi’an, Shaanxi, China

**Keywords:** covered stent, coronary artery perforation, percutaneous coronary intervention

## Abstract

**Background:**

Covered stents are effective in treating coronary artery perforation (CAP), however, the high rate of immediate device deployment failure and in-stent restenosis have limited the application of the currently covered stents.

**Methods:**

We designed a covered stent system consisting of a single layer of drug-eluting stent and a layer of polytetrafluoroethylene (PTFE) membrane wrapped at the outer layer of the stent. The immediate sealing effect of our novel covered stent was observed by using an Ellis type III CAP model. The device’s success was defined as its ability to seal the perforation, assessed by visual estimation and final thrombolysis in myocardial infarction (TIMI) 3 flow. The antiproliferative effect was evaluated in 12 swine, which were randomly assigned to treatment (sirolimus-eluting covered stents) and control (bare metal covered stents) groups. Coronary angiography and optical coherence tomography (OCT) were performed at index procedure, 1- and 6-month after stent implantation. All swine were sacrificed for histopathological analyses at 6-month.

**Results:**

The device success rate was 100%. All swine were alive at 6-month follow-up. At 1-month, the treatment group had a larger minimal luminal diameter (MLD) (1.89 ± 0.29 mm vs. 0.63 ± 0.65 mm, *p* = 0.004) and lower late luminal loss (LLL) (0.47 ± 0.15 mm vs. 1.80 ± 0.34 mm, *p *< 0.001) compared with control group. At 6-month, the treatment group had a numerically higher MLD (0.94 ± 0.75 mm vs. 0.26 ± 0.46 mm; *p* = 0.230) and lower LLL (1.43 ± 0.85 mm vs. 2.17 ± 0.28 mm; *p* = 0.215) compared with control group. Histological analyses revealed the mean plaque area was lower in the treatment group (2.99 ± 0.81 ${\mathrm{mm}}^{2}$ vs. 4.29 ± 0.77 ${\mathrm{mm}}^{2}$, *p* = 0.035) than in the control group. No in-stent thrombosis was observed in either group.

**Conclusions:**

In the porcine model of coronary perforation, the PTFE membrane wrapped sirolimus-eluting stent showed a high device success rate in sealing the perforation. The drug-eluting covered stent demonstrated a relatively sustained antiproliferative effect up to 6 months post-implantation.

## 1. Introduction 

Coronary artery perforation (CAP) is a rare but life-threatening complication of percutaneous coronary intervention (PCI) [1]. Covered stents are considered an effective bailout strategy for CAP [2, 3]. Currently, the most widely used covered stent, GRAFTMASTER stent (Abbott Vascular), is designed in a “sandwich” fashion. A Polytetrafluoroethylene (PTFE) membrane is placed between two layers of 316L stainless steel stents [4]. However, such design has led to a large profile and the flexibility and trackability of the device are compromised. Reports have suggested that the device failure rate was 14.6% in complex lesions, such as calcified and torturous lesions [5]. Moreover, at 3-year follow-up, the incidence of target vessel revascularization (TVR) and definite stent thrombosis post this double-layer cover stents implantation was 26% [6] and 3.5% [7], respectively. To reduce the adverse events and improve the deliverability, newer generation of covered stents, such as BeGraft (Bentley InnoMed GmbH, Hechingen, Germany) and PK Papyrus (Biotronik, Lake Oswego, OR, USA) stents, have adopted the design to a single layer metal stent [8]. In comparison to the GRAFTMASTER, the BeGraft and PK Papyrus were associated with higher device success rates [9]; however, both stents did not lower the incidence of in-stent restenosis (ISR) and TVR [10], as compared with the GRAFTMASTER.

The underlying mechanism of ISR after covered stent implantation has remained elusive. A putative reason is that PTFE, as a foreign body, can stimulate intimal proliferation [11, 12, 13]. To resolve this issue, some covered stents [6] use the pericardium, a collagen-rich biological tissue, to replace the PTFE membrane. Disappointingly, the pericardium-covered stent was not associated with a lower incidence of ISR than that of the PTFE or polyurethane stent [6]. Previous studies [12, 14] showed that the combination of a PTFE-covered stent and an underlying long sirolimus-eluting stent implantation is associated with better angiographic follow-up results, as evidenced by a reduction in the incidence of ISR. Such data indicate that antiproliferative drugs could decrease covered-membrane induced intimal proliferation. Therefore, we designed a covered stent system consisting of a single layer of drug-eluting stent and a layer of PTFE membrane wrapped at the outer layer of the stent. In this study, we aimed to evaluate the safety and efficacy of this drug-eluting covered stent in a porcine coronary perforation model.

## 2. Materials and Methods

### 2.1 Experimental Protocol 

Study animals were swine, 8–9 months old, weighing 25–30 kg. These animals were pretreated with aspirin (100 mg/day) and clopidogrel (75 mg/day) for 5 days prior to the procedure. On the day of the procedure, swine were anesthetized with propofol, and received continuous supplemental oxygen through an oxygen mask. The femoral artery was punctured using the Seldinger method [15]. Throughout the entire procedure, continuous hemodynamic and surface electrocardiographic monitoring were maintained. Heparin (200 IU/kg) was administered via the sheath to achieve activated clotting time >300 seconds. The target coronary artery was engaged using a standard 6-F JR4 guide catheter, and control angiograms of coronary arteries were performed in two orthogonal views [16, 17].

**Fig. 1.**
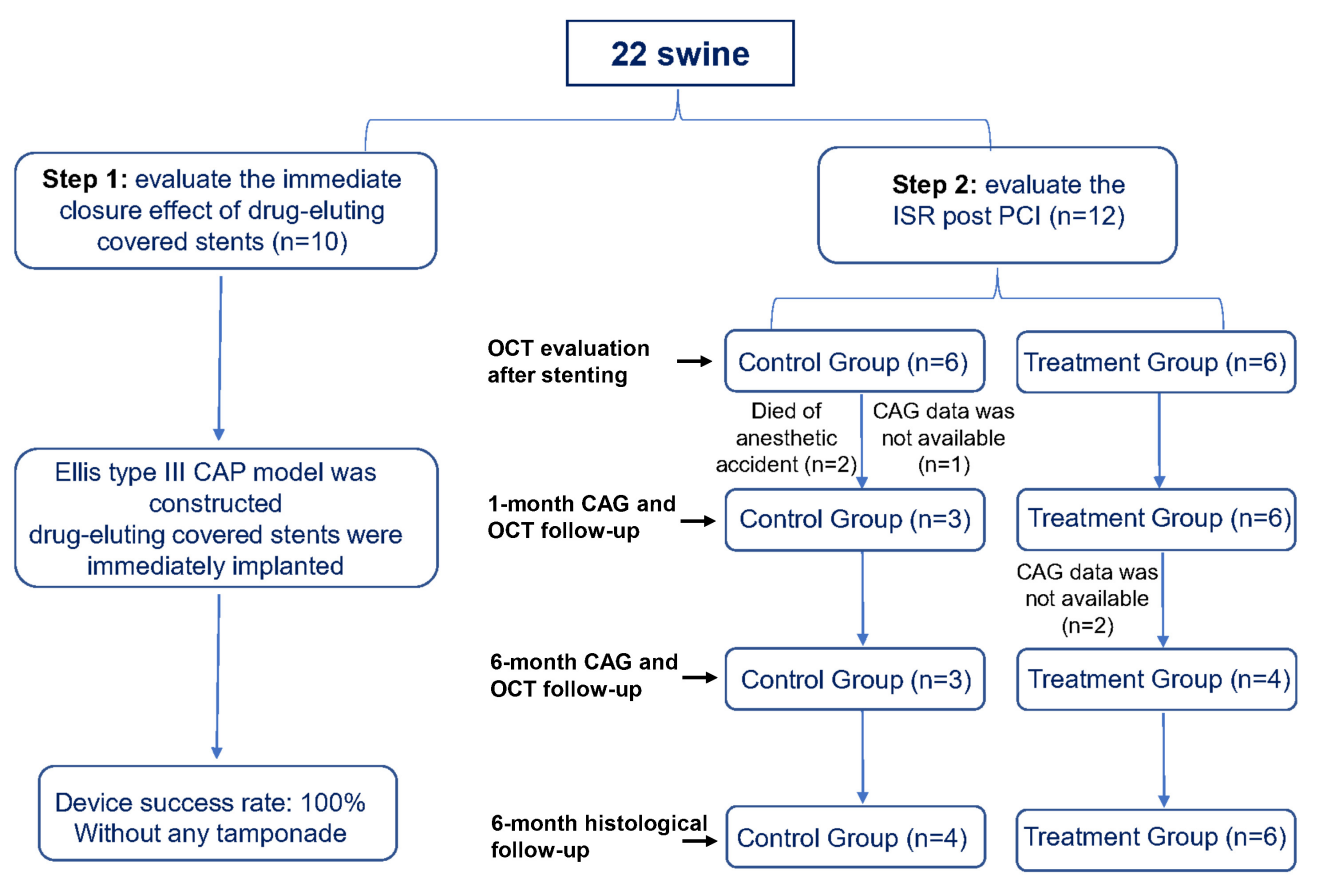
**The flow chart of the study**. CAG, Coronary angiography; CAP, coronary perforation; ISR, in-stent restenosis; OCT, optical coherence tomography; PCI, percutaneous coronary interventions.

Two experimental steps were conducted (Fig. 1). First, to evaluate the immediate closure effect, a CAP model was constructed in 10 swine and subsequently, drug-eluting covered stents were immediately implanted. The success of the device was defined by its ability to seal the perforation, which was assessed by visual estimation and final thrombolysis in myocardial infarction (TIMI) 3 flow [9]. Second, to evaluate the ISR, 12 swine were divided into the treatment (drug-eluting covered stents) and control (bare mental covered stents) groups. In the present study, we recommended the covered stent should be deployed at the nominal pressure of 8 atm for 10–20 seconds. The stent implantation was optimized and evaluated by optical coherence tomography (OCT). Post-dilation with a non-compliant balloon was performed according to the OCT expert consensus recommendations [18]. Coronary angiography and OCT were performed 1- and 6-months post stent implantation. If the stented artery was not occluded, the OCT examination was conducted. Aspirin (100 mg/day) and clopidogrel (75 mg/day) were administered for 6 months. All swine were sacrificed for histopathological analysis at 6-month.

### 2.2 Study Devices

The covered stent system in the present study is a balloon-expandable stent system, consisting of a single layer sirolimus-eluting stent and an expanded polytetrafluoroethylene (ePTFE) membrane wrapped at the outer surface of the stent. The platform of the stent is 316L stainless steel with Poly Lactic-co-Glycolic Acid (PLGA) biodegradable polymer. In the control group, a single layer bare mental without sirolimus-eluting covered stents were used. The covered stent system has a small crimped profile. The crimped profile of the covered stent system used in this study ranged between 1.1 and 1.3 mm. The size of the device implanted in the study was 2.75 mm in diameter and 18 mm in length for all the swine. The dosage of sirolimus on the covered stent was 225 μg.

### 2.3 The Coronary Artery Perforation Model

**Fig. 2.**
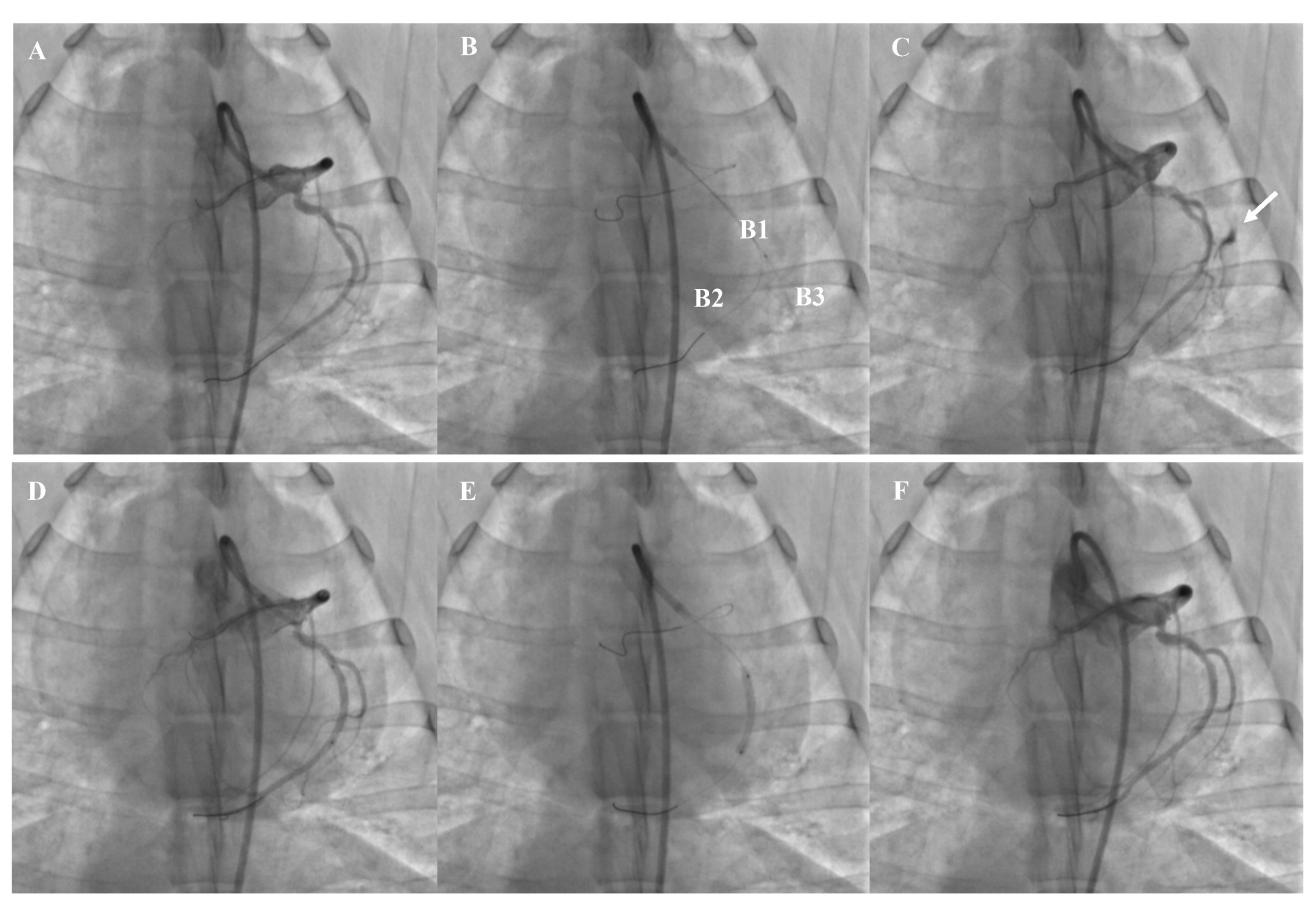
**The representative example of CAP model and covered stent implantation**. (A) Baseline CAG. (B) Construction of CAP model (B1, micro-catheter, B2, soft guidewire, B3, stiff guidewire). (C) Streaming of contrast through an exit hole (﻿white arrow). (D) Stent positioning. (E) Stent dilation. (F) Complete closure of the perforation. CAP, coronary perforation; CAG, Coronary angiography.

Ellis type III CAP model, defined as frank streaming of contrast through a >1 mm exit hole [19], was created in the present study. As shown in Fig. 2, after engaging 6F JR 4 guiding catheters, a guidewire with soft tip was placed at the distal left anterior descending coronary artery (LAD) for device deployment. A stiff guidewire assisted by the micro-catheter was used to pierce the coronary artery and create perforation. All the CAP models were made in LAD, and the middle of LAD (without any branches) was preferred to be pierced to create perforation. If the perforation was not created successfully by the stiff guidewire, the micro-catheter was advanced through the wire to create a perforation. A successful perforation was confirmed by coronary angiography.

Loaded with the soft tip guidewire, the covered stent was released using 8–12 atm single expansion for 5–10 s to seal the perforation. Angiography was repeated to ensure that the perforation was completely occluded (Fig. 2). OCT was used to determine whether any post-dilation was needed and to ensure that no serious dissection or thrombosis occurred. Finally, the femoral sheath was withdrawn, and the puncture site was compressed for 20–30 min to stop the bleeding. An intramuscular injection of penicillin at a dose of 8 × ${\mathrm{10}}^{4}$ U/kg was administered to prevent infection.

### 2.4 Quantitative Coronary Angiography (QCA) Analysis

The Medis QCA-CMS 6.0 system (Raleigh, NC, USA) was used for quantitative coronary angiography (QCA) measurements. In this study, the following parameters were included: reference luminal diameter (RLD) at preimplantation, minimal lumen diameter (MLD), late lumen loss (LLL), diameter and area of stenosis at post-implantation and follow-up. The stenosis diameter was calculated as $\left[1-\frac{M\,L\,D}{\text{ reference vessel diameter }}\right]$ at explant. The percent diameter restenosis was calculated by ((RLD-MLD)/RLD) × 100. The LLL was calculated as [MLD at post implantation – MLD at follow-up].

### 2.5 Optical Coherence Tomography (OCT) Analysis

OCT was performed using a commercially available C7 OCT system (St Jude Medical, St Paul, MN, USA). OCT was used to optimize the stent implantation and evaluate the safety index such as any incidence of dissection and in-stent thrombosis.

### 2.6 Histological Analysis

Stented segments of the coronary arteries were fixed with 10% formalin, dehydrated in a graded series of ethanol and embedded in polymethyl methacrylate. After polymerization, 5 sections were sliced from the proximal to distal portion in each stented segment. Sections were stained with methylene blue and eosin, and examined by light microscopy. The plaque area was calculated by the Image J software. The mean plaque area derived from 5 sections was compared between the treatment and control groups.

### 2.7 Statistical Analysis 

Variables with normal distribution are expressed as the mean ± standard deviation (SD), while variables with skewed distribution are expressed as the median with interquartile range. Means of 2 continuous variables were compared by independent student’s *t*-test or Mann-Whitney U test when appropriate. The frequencies of categorical variables were compared using Fisher’s exact test. *p* values < 0.05 were considered statistically significant. All statistical analyses were performed with SPSS 25.0 (SPSS Inc., IBM Corp., Chicago, IL, USA).

## 3. Results

### 3.1 The Construction of the CAP Model and Closure Effect of Covered Stents

The CAP model was successfully developed in all 10 swine. In experimental step 1, all covered stents were successfully deployed, and the device success rate was 100% without any incidence of tamponade. A representative case showing the development of the CAP model and subsequent sealing of the perforation by a covered stent is presented in Fig. 2.

### 3.2 QCA Analysis

**Fig. 3.**
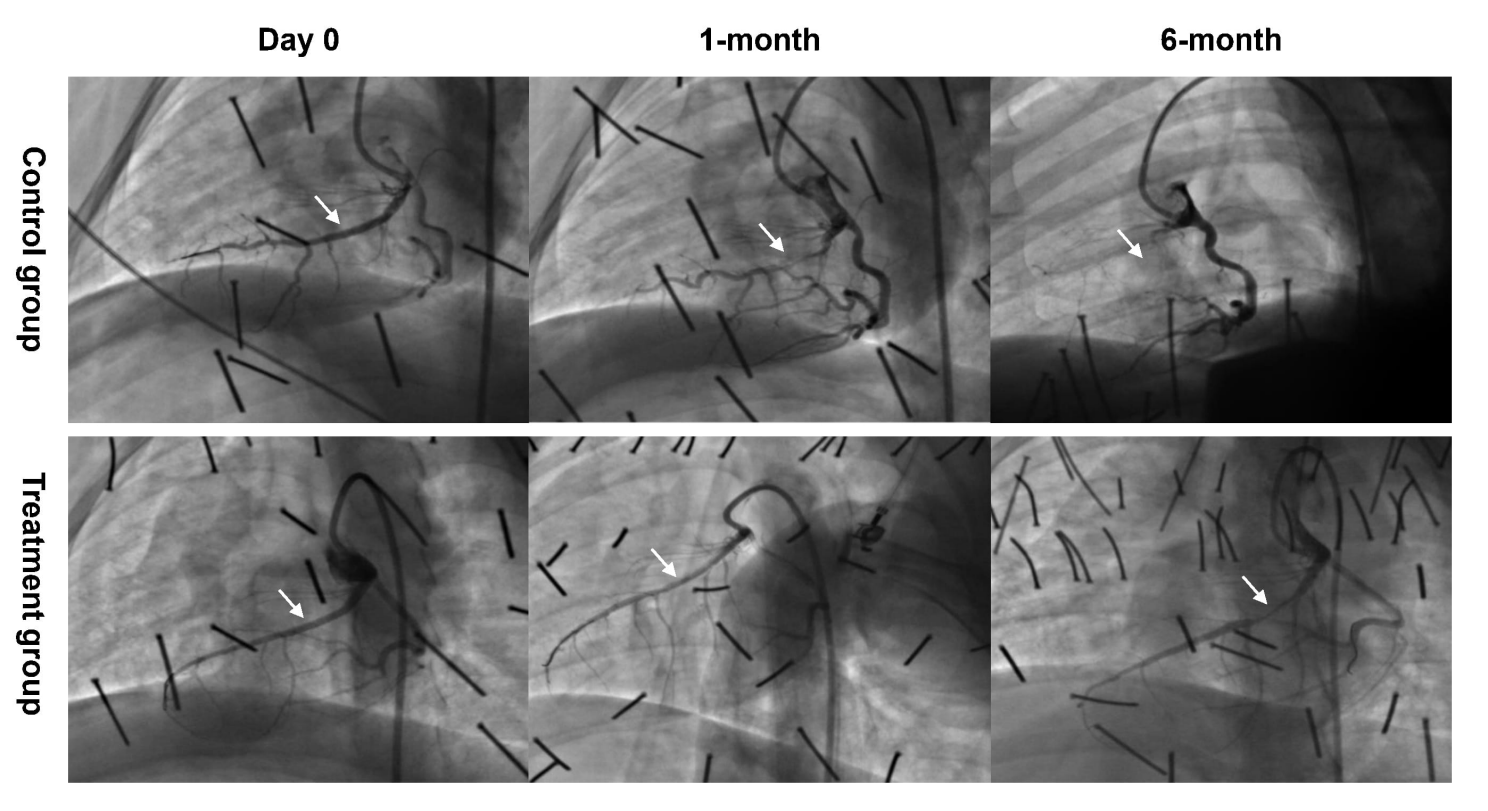
**Representative examples of CAG follow-up at all time points**. Two swine died of anesthetic accident in the control group. The CAG data was not available in 1 swine from the control group and 2 swine from the treatment group, respectively. CAG, Coronary angiography.

Among the 12 swine, two died of anesthetic accident in the control group. As shown in Table 1, the RLD was similar between the treatment and control groups (2.59 ± 0.19 mm vs. 2.63 ± 0.26 mm, *p* = 0.549). At 1-month follow-up, the CAG data was not available in 1 swine from the control group. The treatment group had a much larger MLD (1.89 ± 0.29 mm vs. 0.63 ± 0.65 mm, *p* = 0.004) and lower LLL (0.47 ± 0.15 mm vs. 1.80 ± 0.34 mm, *p *< 0.001) than those in the control group. The percentage area of stenosis (AS%) (45.56 ± 12.86 vs. 90.35 ± 11.51, *p* = 0.001) and diameter of stenosis (DS%) (26.69 ± 9.19 vs. 75.70 ± 23.69, *p* = 0.002) were much lower in the treatment group than those in the control group. At 6-month follow-up, the CAG data was not available in 2 swine from the treatment group. In comparison to the control group, the treatment group had a numerically increased MLD (0.94 ± 0.75 mm vs. 0.26 ± 0.46 mm; *p* = 0.230), decreased LLL (1.43 ± 0.85 mm vs. 2.17 ± 0.28 mm; *p* = 0.215), decreased AS% (79.29 ± 22.08 vs. 97.31 ± 4.65, *p* = 0.232) and decreased DS% (62.53 ± 29.82 vs. 90.54 ± 16.39, *p* = 0.207). Representative images are presented in Fig. 3.

**Table 1.** **Angiographic data at all time points**.

	Control group	Treatment group	*p* value
Day 0			
	N = 4	N = 6	
RLD, mm	2.63 ± 0.26	2.59 ± 0.19	0.549
MLD, mm	2.52 ± 0.25	2.44 ± 0.22	0.200
1-month			
	N = 3	N = 6	
MLD, mm	0.63 ± 0.65	1.89 ± 0.29	0.004
LLL, mm	1.80 ± 0.34	0.47 ± 0.15	<0.001
DS%	75.70 ± 23.69	26.69 ± 9.19	0.002
AS%	90.35 ± 11.51	45.56 ± 12.86	0.001
6-month			
	N = 3	N = 4	
MLD, mm	0.26 ± 0.46	0.94 ± 0.75	0.230
LLL, mm	2.17 ± 0.28	1.43 ± 0.85	0.215
DS%	90.54 ± 16.39	62.53 ± 29.82	0.207
AS%	97.31 ± 4.65	79.29 ± 22.08	0.232

RLD, Reference Luminal Diameter; MLD, Minimal Luminal Diameter; LLL, Late Luminal Loss; DS, Diameter Stenosis; AS, Area Stenosis.

### 3.3 OCT Analysis

**Fig. 4.**
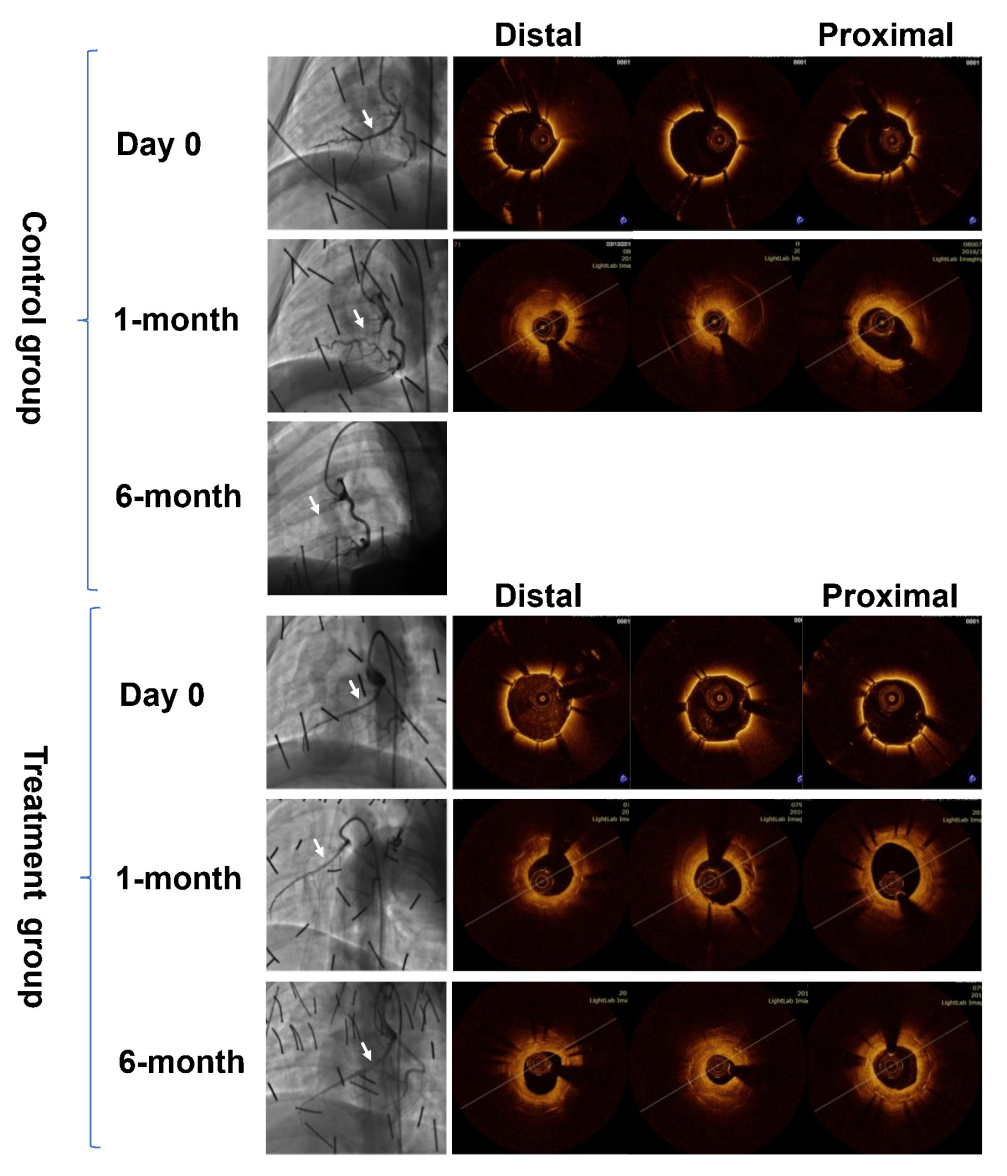
**Representative examples of OCT follow-up at all time points**. OCT, Optical Coherence Tomography.

No in-stent thrombosis was observed for all the available cases in any group at 1-month and 6-month OCT follow-up. Representative examples of such cases undergoing control and drug-eluting covered stents implantation are presented in Fig. 4. According to OCT analysis, at 1-month and 6-month follow-up, complete endothelialisation on the stents were observed for all the available cases in both groups.

### 3.4 Histological Analysis

**Fig. 5.**
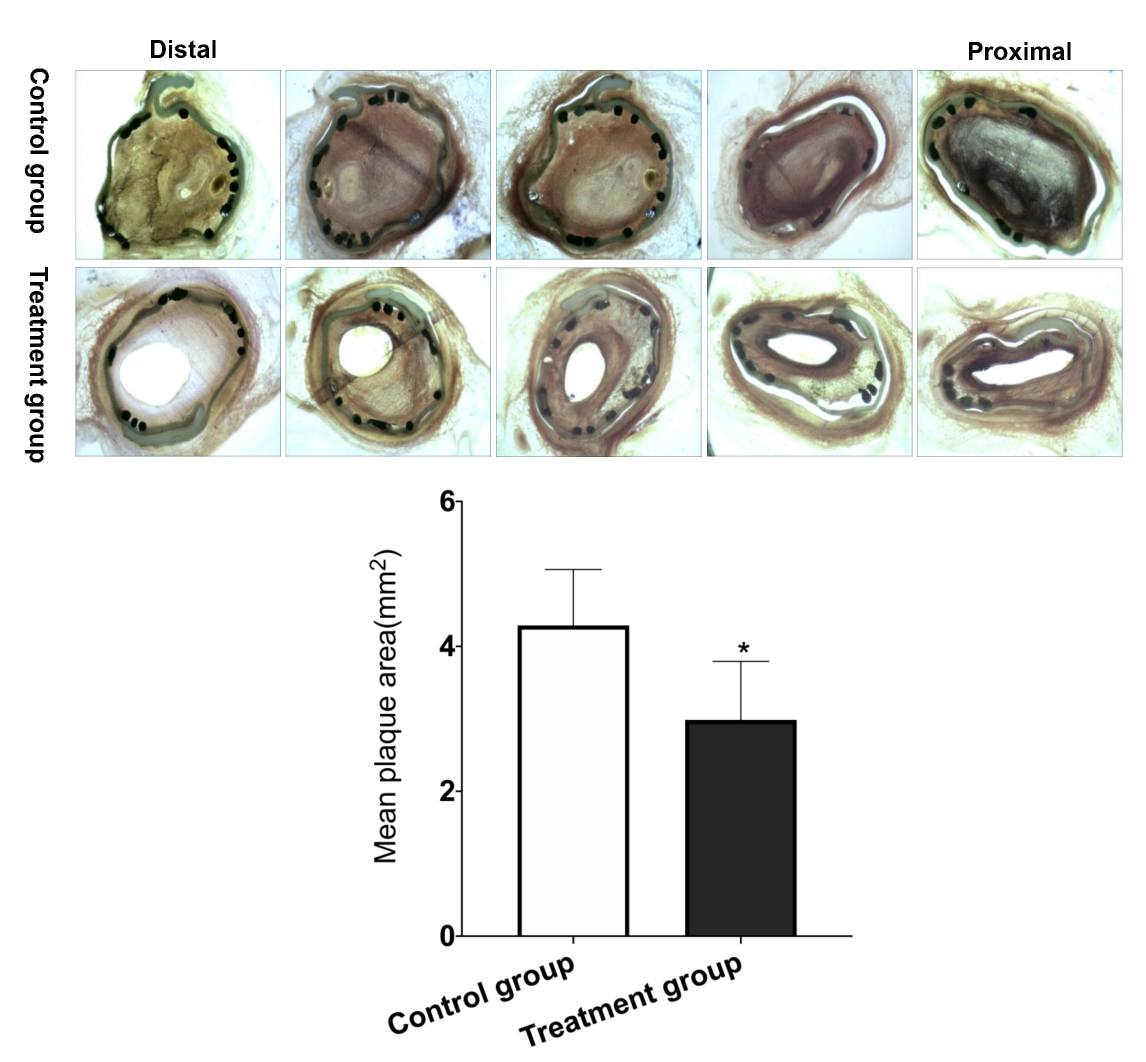
**Representative examples of histological follow-up**. *p < 0.05 Treatment group (n = 6) vs. Control group (n = 4).

All the swine underwent histological analysis. Histomorphometric analysis results are shown in Fig. 5. At 6-month follow-up, histopathological analysis showed complete endothelialisation for all the cases in both groups (n = 4 in the control group and n = 6 in the treatment group). The mean plaque area was lower in the treatment group than in the control group (2.99 ± 0.81 ${\mathrm{mm}}^{2}$ vs. 4.29 ± 0.77 ${\mathrm{mm}}^{2}$, *p* = 0.035).

## 4. Discussion

In the present study, we investigated the safety and efficacy of a novel covered stent in a swine coronary perforation model. The findings from the present study are summarized as follows: (1) A controllable and safe CAP model was developed using guidewire assisted by micro-catheter. (2) The novel drug-eluting covered stent had a high success rate in sealing the coronary perforation without any incidence of cardiac tamponade. (3) The novel drug-eluting covered stent demonstrated a relatively sustained antiproliferative effect up to 6 months post implantation.

Coronary artery perforation (CAP), especially Ellis type III CAP, is a life-threatening complication of PCI, and is associated with a high incidence of cardiac tamponade (42.9%) [19], however, the technique of creating an animal model of CAP has not been previously addressed. To evaluate the efficacy of covered stents, we developed a CAP animal model. Our model has two salient features. First, the perforation was controllable. We used a micro-catheter to control the position and size of the perforation. Second, the model was relatively secured. A protective soft tip guidewire allowed an immediate deployment of the covered stent, thereby avoiding cardiac tamponade and percutaneous pericardial drainage. In this study, all Ellis type III perforations were successfully established using guidewire guided by micro-catheter without inducing any cardiac tamponade required treatment. We believe this coronary perforation model is promising for a variety of applications such as vascular injury, cardiac tamponade, and device evaluation in cases with coronary perforation.

Covered stents are considered an effective bailout strategy for Ellis types III coronary perforation which cannot be salvaged with a prolonged balloon inflation [5]. Covered stents with a “sandwich-like” structure have a device failure rate in the range of 16.7% [20] to 23.8% [21] in complex lesions. Compared to the GRAFTMASTER stent, the new generation PK Papyrus stent is associated with a shorter delivery time (8 vs. 15 min, *p* = 0.001), and a higher procedural success rate (86% vs. 69%, *p* = 0.216) [9]. Similar to the BeGraft and PK Papyrus stents, the covered stent established in the present study adopted the design of a single layer metal stent with a single layer of PTFE membrane, and has a small crimped profile that ranged between 1.1 and 1.3 mm. In our study, the device success rate was 100% without any incidence of cardiac tamponade and cardiac arrest.

In-stent restenosis (ISR) is a major concern of covered stents that needs to be resolved. Current data suggest that the rate of ISR in PTFE covered stent-treated patients range between 29.2% [20] and 54.6% [22]. Although the deliverability of the newer generation covered stents has greatly improved due to the adoption of a single layer design of metal stent, the incidence of ISR remains high. Kufner *et al*. reported outcomes from 61 coronary perforation patients treated with the BeGraft covered stent. During a follow-up of 192.8 ± 144.2 days, the incidence of binary angiographic restenosis was 26.9% in overall patients and 45.5% in patients with very complex lesions [23].

The underlying mechanism of ISR post PTFE covered stent implantation is considered to be associated with the following reasons [11, 12, 13, 21]: (1) delayed endothelialization, (2) thrombus formation, (3) intimal proliferation stimulated by PTFE, and (4) stent malapposition or vascular injury such as dissection. To avoid such causes of ISR, intensive antiplatelet treatment, OCT or IVUS guided stent implantation and specific histocompatible materials could be used. To improve the biocompatibility, the asneugraft® Dx stent used pericardium, a collagen-rich biological tissue, to replace the PTFE membrane. Unfortunately, the pericardium covered stent demonstrated a higher occurrence of ISR than the PTFE covered stent [6].

Papafaklis *et al*. [14] and Hou *et al*. [12] reported that the combination of PTFE-covered stents and an underlying long sirolimus-eluting stent implantation offered better angiographic follow-up results, as evidenced by a decrease in the stent-edge or stent-segment binary restenosis. Therefore, we hypothesized that antiproliferative drugs could decrease neointimal proliferation induced by the PTFE. In the present study, we used a stent platform with PLGA biodegradable polymer. The dosage of sirolimus on the covered stent was 225 μg, which may decrease the restenosis. Furthermore, OCT was introduced to optimize the stent implantation and avoid any vascular dissection, a major risk factor of ISR [24]. At 1-month angiographic follow-up, the LLL was significantly lower with our novel drug-eluting covered stent than with the non-drug eluting stents. At the 6-month follow-up, in comparison to the control covered stent, the drug-eluting covered stent still demonstrated a lower LLL. Histological analysis showed that, in comparison to the control stent, the drug-eluting covered stent significantly decreased the mean plaque area. The drug-eluting covered stent demonstrated a relatively sustained antiproliferative effect up to 6 months post implantation. Notably, the MLD reduced gradually after post PCI in both groups (Table 1). Although restenosis is a progressive phenomenon that begins in the early hours after the barotrauma determined by PCI [25], and even after DES implantation, the rate of restenosis is still around 10% [26]. Nevertheless, our results could provide a new conceptual design of covered stent to treat neointimal proliferation induced by the covered membrane. However, these results were obtained in a porcine model, and as such, the safety and efficacy of this drug-eluting covered stent needed to be further investigated in human subjects. The in-stent thrombosis is a safety concern associated with covered stent implantation. In the present study, OCT was introduced to avoid stent malapposition or under expansion, a risk factor for thrombosis. At 1-month follow-up, no in-stent thrombosis was observed, and all stent segments demonstrated complete endothelialization in swine examined with OCT.

Covert stent was mainly used for the treatment of CAP, but also could be used for other indications such as coronary artery aneurysms (CAA), pseudoaneurysms, arterial-venous fistula, etc. [27, 28, 29]. Previous studies showed that CAA was the second most frequent indication for covered stents, but with a relatively high rate of stent thrombosis [2, 27]. Using intravascular imaging or a computed tomography scan to achieve accurate landing zone assessment and stent sizing during CAA with covered stents is recommended [27]. Post-dilatation and procedural guidance with intravascular imaging may lead to optimal apposition and expansion of the stent, which could improve the long-term outcomes. Besides CAP and CAA, covert stent also could be used to treat pseudoaneurysm, arterial-venous fistula, saphenous vein graft, etc. [27, 28, 29, 30]. Information regarding covered stents in these off-label use was heterogeneous and limited to small sample size studies, highlighting the unmet need for large-scale trials in these settings.

### Study Limitations 

First, we used porcine coronary arteries with no atherosclerosis, calcification, or tortuous lesions. However, in clinical cases, covered stents are usually used to treat perforation in the atherosclerotic and calcified arteries. Therefore, the results should be interpreted with caution. Second, during the follow-up, the CAG data was not available in 3 swine, which decreased the sample size that was used to evaluate CAG results. However, these 3 swine underwent histological analysis. Third, pharmacokinetic studies on the release of sirolimus were not conducted. In addition, optimal antiplatelet treatment duration after covered stent implantation has not been well studied, and no specific recommendations are available. In real-life scenarios CAP often occurs in patients without or delayed dual antiplatelet therapy due to the fear of recurrent bleeding. In our study, no in-stent thrombosis was observed for all the available cases which could be possibly explained by the dual antiplatelet therapy administration during the whole study period. Therefore, our findings should not be extrapolated to a real-world setting and should be confirmed in future studies. Finally, the underlying mechanisms of the antiproliferative effect were not investigated. However, our study provides preliminary data on a novel drug-eluting covered stent that warrants further investigations.

## 5. Conclusions

In the present study, the swine coronary perforation mode which was developed using guidewire assisted by micro-catheter seemed to be a controllable and safe CAP model. In this CAP model, implantation of a novel drug-eluting covered stent was proven to be safe and effective with a high device success rate and without any incidence of stent thrombosis or delayed endothelialization. Moreover, the novel drug-eluting covered stent demonstrated a relatively sustained antiproliferative effect up to 6 months post implantation. Our findings need to be confirmed in future studies with a larger sample size and long-term follow-up.
